# An improved human activity recognition technique based on convolutional neural network

**DOI:** 10.1038/s41598-023-49739-1

**Published:** 2023-12-19

**Authors:** Ravi Raj, Andrzej Kos

**Affiliations:** Faculty of Computer Science, Electronics, and Telecommunications, AGH University of Science and Technology, Aleja Adama Mickiewicza 30, 30-059 Krakow, Poland

**Keywords:** Energy science and technology, Engineering

## Abstract

A convolutional neural network (CNN) is an important and widely utilized part of the artificial neural network (ANN) for computer vision, mostly used in the pattern recognition system. The most important applications of CNN are medical image analysis, image classification, object recognition from videos, recommender systems, financial time series analysis, natural language processing, and human–computer interfaces. However, after the technological advancement in the power of computing ability and the emergence of huge quantities of labeled data provided through enhanced algorithms, nowadays, CNN is widely used in almost every area of study. One of the main uses of wearable technology and CNN within medical surveillance is human activity recognition (HAR), which must require constant tracking of everyday activities. This paper provides a comprehensive study of the application of CNNs in the classification of HAR tasks. We describe their enhancement, from their antecedents up to the current state-of-the-art systems of deep learning (DL). We have provided a comprehensive working principle of CNN for HAR tasks, and a CNN-based model is presented to perform the classification of human activities. The proposed technique interprets data from sensor sequences of inputs by using a multi-layered CNN that gathers temporal and spatial data related to human activities. The publicly available WISDM dataset for HAR has been used to perform this study. This proposed study uses the two-dimensional CNN approach to make a model for the classification of different human activities. A recent version of Python software has been used to perform the study. The rate of accuracy for HAR through the proposed model in this experiment is 97.20%, which is better than the previously estimated state-of-the-art technique. The findings of the study imply that using DL methods for activity recognition might greatly increase accuracy and increase the range of applications where HAR can be used successfully. We have also described the future research trends in the field of HAR in this article.

## Introduction

In recent decades, the application of Artificial intelligence (AI) techniques has been rapidly growing in almost every field including surveillance, healthcare, industries, space exploration, and many more^1,2^. The integration of AI technology in each field of research and study has been enhanced rapidly, which is the major reason behind the attention of researchers and technologists toward this technique. Pattern recognition is an important and widely used area of computer vision. Classification of images is the process by which images can be categorized into one of multiple known or predefined classes. Machine learning (ML) and deep learning (DL) are subsets of AI^1,2^. ML and DL might be implicated during the classification of images to get better quality outcomes. The techniques of ML are becoming the most important part of computer vision systems. For the reduction of input data dimension into a better controllable format by keeping only nonredundant and informative sections, a feature extraction process is required. Extraction and classification of the feature are the major steps of almost every ML task. Extraction of handcrafted features is carried out in the traditional ML algorithms. Although, most of the researchers are focusing on automatic learning, extraction, and classification of features to enhance the accuracy and potential of intelligent systems. HAR is an area of research within the larger fields of AI and ML, with an emphasis on creating methods and models to recognize and categorize human activity using information gathered from several sensors.

**Figure 1:**
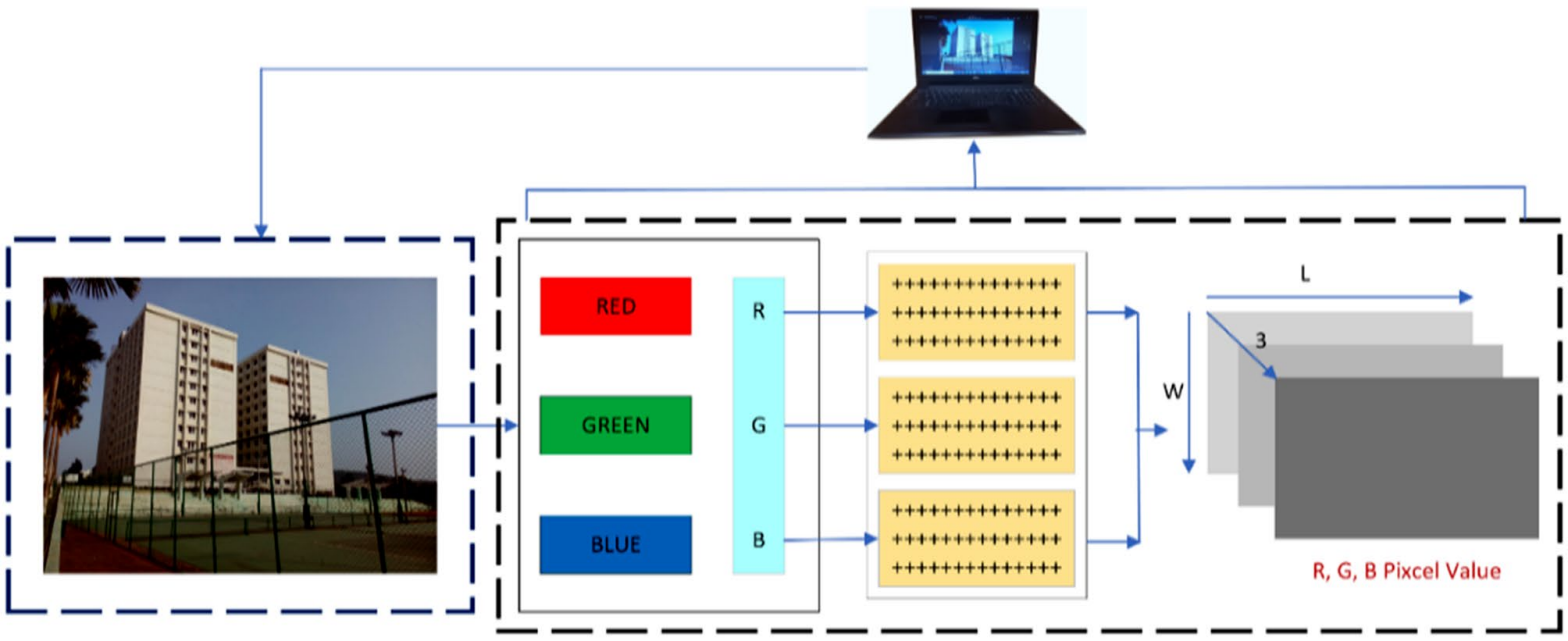
Process of image recognition in a computer.

Recognizing and classifying human actions and behaviors using information often collected by cameras, sensors, or additional tools is the main objective of the computer vision and ML discipline known as HAR. HAR aims to automatically recognize and interpret what an individual is doing at a particular instant using the provided information^3^. HAR has many applications, including medical diagnosis, sports assessment, surveillance, human-computer interface, and many more. HAR is an area of study and research of image classification that belongs to the instantaneous recognition of activities done by humans based on the recordings done by the time series utilizing sensors. CNNs are used in HAR because of their capacity to automatically extract complex characteristics from unprocessed data from sensors, thereby removing the requirement for human feature extraction. Figure 1 describes the complete process of image recognition by a computer. This process starts with the breakdown of an image into the channels of three colors red (R), green (G), and blue (B). That means input data must be converted into R, G, and B, first. In the second step, these color channels are converted to the image pixels and finally, the computer can recognize every pixel value and understand the size of the image.

In assignments involving pattern recognition, the classification of the image is an essential problem. It builds the background for other tasks of computer vision or pattern recognition such as segmentation, localization, and detection^4^. Pattern recognition has significance mainly because it makes it possible to collect useful data, automate operations, generate decisions based on facts, enhance safety, improve technology, enhance medical services, recognize cognitive operations, ensure performance, and promote science in various fields including environmental science, business, natural language processing, and many more. Generally, an approach of the dual-stage was utilized to resolve the problems of image classification in the past. Firstly, the custom-crafted features were extracted by utilizing the feature descriptors from images, and these features worked as classifier input data for a classifier that has the ability to be trainable in the system. The prime obstruction of this method was in the classification experiment accuracy, which was generally based on the outline of the stages of the feature extraction, which was normally validated to be a vigorous problem^5^. Inertial or motion sensors such as accelerometers and gyroscopes generally found in smartphones and smartwatches would evaluate movement characteristics such as angular velocity and acceleration of the human body and utilize them to understand the competency of HAR models. There are a lot of developments that have been formed into integrated sensing technologies such as edge computing, sensors, cloud, and IoT. Where, Sensors are very reasonably cost electronic devices, and these sensors could be smartly embedded or integrated with non-portable and portable equipment, thus most of the HAR-related experiments are utilizing sensors.

Nowadays, DL is the most important subset in the field of ML. Neural network design, design of features, requirements for data, computing power, accessibility, and execution situations are the main areas where ML and DL differ. While DL is a particular subset of ML that is concentrated on ANNs and automated feature extraction, ML collectively is a broader discipline that includes a variety of techniques. In the recent decade, DL attained outstanding performance in pattern recognition and computer vision tasks, with various utilizations such as object detection, classification of characters or images or objects, restoration of super-resolution, etc.^6^. According to the type of training set, processes of learning might be carried out in unsupervised, semi-supervised, or supervised ways. Also, reinforcement learning is a special kind of procedure of learning technique that is generally described in the case of semi-supervised and a few cases under the approaches of unsupervised learning^7^. While supervised learning gives excellent outcomes, in the case of training samples with insufficient labeled data such as in the human activity recognition system, other approaches of DL might be implied for the extraction of features from unlabeled images.

In the last decade, sensor-based HAR gained popularity as a study issue, with researchers first implementing conventional ML techniques for HAR tasks: sensor-based movement data collection, preprocessing of data, action segmentation, extraction of features, and activity classification^8^. Classical ML techniques, including decision trees^9^, support vector machines (SVM)^10^, random forests^11^, and Bayes^12^, have shown outstanding effectiveness in the classification of activities. However, ML has several limitations and mainly depends on manual feature extraction because of its simple learning procedure. Manually extracting features typically relies on careful decision-making based on user experience and expertise in the topic, including statistical and frequency range features. Furthermore, the characteristics of handcraft are limited to being used to characterize certain basic human actions rather than complex ones. Therefore, it proves difficult for simple ML methods to adapt to novel, complex HAR situations^13^. End-to-end ANN has enabled DL to accomplish autonomous feature extraction, significantly reducing the laborious and time-consuming process of human feature extraction and automating the complex feature design technique^14^. DL approaches, which have improved effectiveness and accuracy in classification, are now widely used in HAR and are efficient HAR techniques. Recurrent neural networks (RNN) and CNN are examples of standard ANNs. CNN is a feed-forward neural network that contains characteristics like weight exchange, local connection, and down-sampling and performs exceptionally well in computer vision. In the disciplines of image annotation, sequence annotation, etc., RNN is frequently employed as a solution to the unsolved issues of variable-length sequencing and dependency over long distances in patterns that appear in feedforward neural networks. RNN is also used for the classification of human activities^15^. Compared with RNN, CNN has better performance in capturing spatial features. DL approaches still have many issues, including the extraction of features, the characterization of features, the classification of features, the accuracy of the recognition system, and the timing of performing recognition tasks. Despite DL's benefit of automatically gathering data features, multiple network architectures have varying degrees of feature characterization ability. Additionally, the patterns in the HAR operation time series are difficult to classify and have both forward and backward significance^16^. For better feature extraction and higher accuracy, we propose a 2D CNN-based HAR. In this study, we are using a fully connected layer that accelerates the training of models and improves classification accuracy. When we tested the proposed model against the reference WISDM dataset, our model performed better.

This article is further divided into eight parts in the following ways: Section "Convolutional neural networks" describes the CNNs in a comprehensive way which includes the description of convolution and pooling layers, Sect. "The architecture of the CNN model for image classification" describes the framework of CNN architecture for the classification of images, in Sect. "Human activity recognition" human activity recognition system has been discussed, Sect. "Research motivation and contribution of the research work" contains the research motivation and contribution of the research work, Sect. "Related work". reviews most important articles related to the human activity recognition by using CNNs, in Sect. "Proposed methodology" we have analyzed a study for the classification of different human activities by using 2D CNN model, Sect. "Experimental result and discussion" describes result and discussion, and Sect. "Conclusion and future perspectives" describes some concluding points and future research trends of this research work.

## Convolutional neural networks

**Figure 2:**
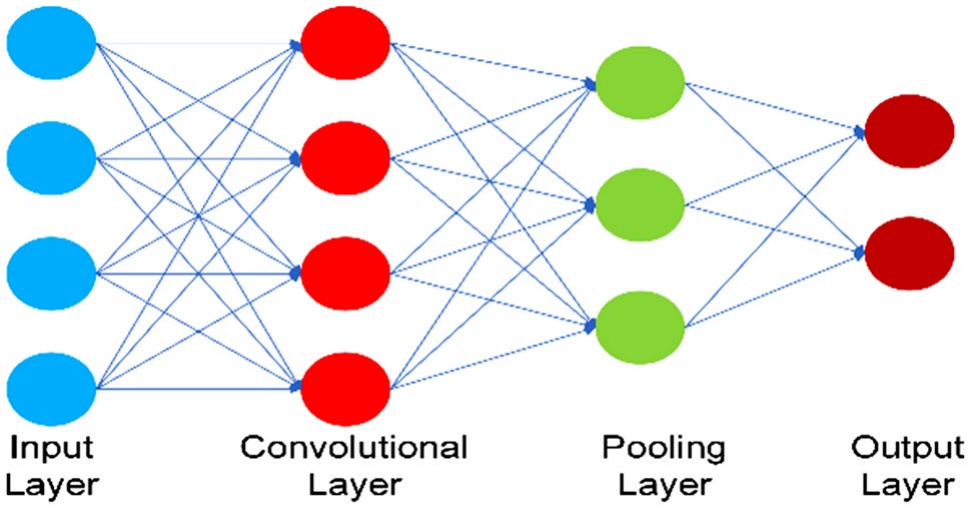
An internal structure of a typical Convolutional Neural Network.

In the 1990s, CNNs were presented, although, because of the limited capacity of computational power and processing potential and, as well as the unavailability of explicated trainable input data, they became out of trend. CNNs are a subset of ANN models based on DL that are mainly developed for the extraction and evaluation of visual data, including pictures and videos. CNNs are very good at activities including image generation, object classification, image classification, hand-gesture recognition, leaf-disease recognition, medical image recognition, facial recognition, and many more^17–20^. CNN structure and working principle are inspired by the structure and operation of the visual system of humans, which is very good at pattern recognition and feature extraction in visual data. CNNs are an effective tool for different tasks because they can automatically learn hierarchical characteristics using unprocessed data. In recent years, CNNs have developed so fast due to the availability of extensive datasets. The model of handmade feature extraction has become outdated in the traditional area of computer vision systems due to advancements in CNN. It is better than previous methodologies because of having an intelligent classification process and classical regression. The most important benefits of CNNs are programmed feature extraction utilizing hierarchical characterizations, convolutional layers utilized for weight sharing, and pooling operations utilized for spatial invariance^21^. CNN, also known as ConvNet, is one of the widely utilized techniques of DL in tasks related to computer vision or pattern recognition. ConvNet is considered the mechanism of human visual perception and incorporates inconsistent convolutional and pooling layers. Generally, the extraction of features is carried out first, and after that, they are configured with a multilayer perceptron (MLP) for regression and classification works. This paper exclusively explains the role of CNN in the classification of images by taking into consideration the estimation of the different activities of human beings in their daily life. CNN has various implications in natural language processing, video, and image recognition, and recommendation systems. By enabling CNNs to automatically acquire hierarchical attributes, reduce the number of parameters, optimize predictions, and improve the efficiency of computation, programmed extraction of features by employing convolutional layers, weight transmission, and pooling actions improves their effectiveness. Figure 2 shows CNN with its different parts such as the input, convolutional, pooling, and output layers.

### Convolutional layers

The convolutional layer works as the extractor of features, and therefore they understand the representation of features from their input data. The convolutional layer has neurons that are structured into the map of the features. The region in the input that generates the feature is known as the receptive field within CNN. Every neuron has a receptive field in the feature map, which is interconnected to a neuron’s neighborhoods in the preceding layer under a collection of the trainable weights, occasionally considered as a filter bank. The input data are convoluted with the learning weights to evaluate a novel map of features and the convoluted outcomes are transmitted along an activation function, which has a nonlinear shape^22^. The weights contained by every neuron inside a map of the feature are obliged to be uniform; although disparate maps of features inside similar convolution layers have disparate weights, various features might be extracted at every position. More generally, the *k*-th output for the *Y*_*k*_ map of feature could be evaluated as Eq. (1)^23^.

1 $$ Y_{k} = \, f\,\left( {W_{k} * x} \right), $$

where the input image is the x; the convolutional filter is the *W*_k_ associated with the *k-*th map of the feature; the two-dimensional operator of the convolutional layer is denoted by the sign of multiplication, which is utilized to evaluate the filter model inner product at every position of the input data; and non-linear activation function is denoted by *f* (*.*). The non-linear features can be extracted with the help of nonlinear activation functions. It enhances the model's abstractions or adaptations to a range of data and improves output divergence.

### Pooling layers

The main important role of the pooling layer is to transform the representation of joint features into a more convenient and usable one that keeps essential information while removing irrelevant information. The spatial resolution can be reduced by using pooling layers in the features map for getting spatial invariance into the input translations and distortions^24,25^. Previously, it was utilized for average aggregation pooling layers to send the image input data from one layer to the next layer. In recent days, it has been used in the defined receptive fields to propagate the optimal value of input images to the next layers^26,27^. The pooling region within a CNN structure is a local window that flows over the mapping of features, enabling the neural network to minimize the input, add spatial invariance, and choose significant features. In CNNs, the traditional pooling techniques are of two types, including average and maximum pooling. Generally, optimal or maximum pooling gathers the biggest element within every receptive area, which is demonstrated by Eq. (2)^23^.

2 $${Yk}_{ij} =\underset{\left(P,q\right)\varepsilon {R}_{ij}}{max}{X}_{kpq}$$

where the pooling operator output for the kth future map is denoted by *Y*_*kij*_. The element at (*p, q*) is *X*_*kpq*_ around the *R*_*ij*_, which is a pooling region, that illustrates a local region around the location (*i, j*).

**Figure 3:**
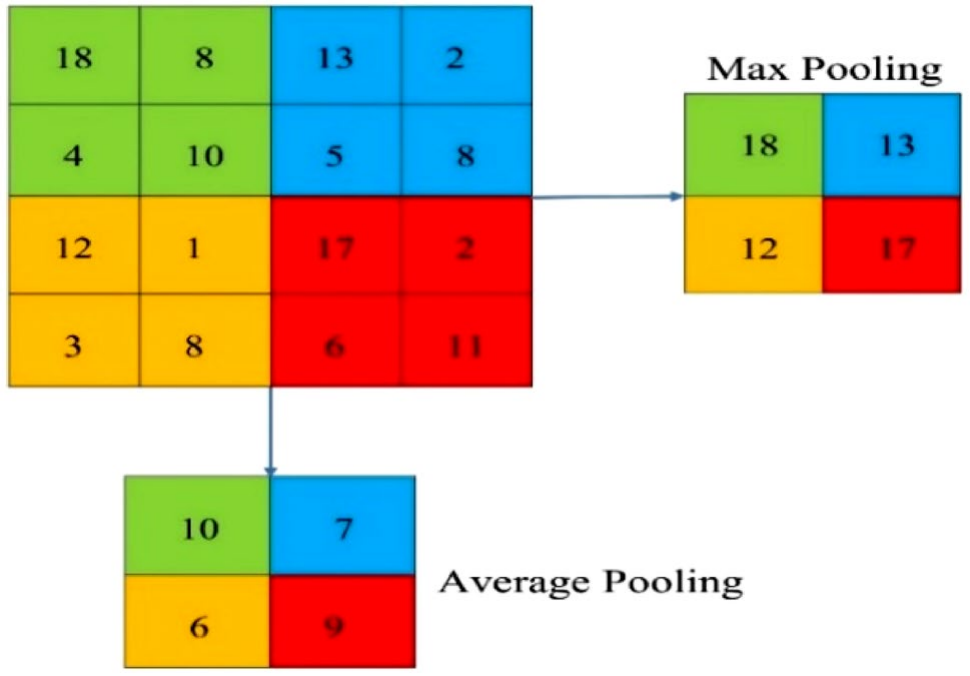
Max vs. average pooling.

Figure 3 demonstrates the difference between average pooling and max pooling. Let’s consider a 4×4 size input image if a filter of 2×2 and two strides is implied, the outputs of max pooling are the optimal value of every region of 2×2, while the outputs of average pooling are the value of the average rounded integer of every region of subsampled.

### Fully connected layers

The model of CNN ends with a fully connected layer. Each neuron of the adjacent layer is connected to every neuron of the previous layer; thus, it is called a fully connected layer. It operates according to the fundamental principles of the common MLP neural network^28^. It is simply a feed-forward neural network. The input data for the fully connected layers is output coming from the pooling layers. The input data is compiled in this layer to provide output. The prior high-level features of nonlinear combinations are implied to be learned by the fully connected layers. Many layers of convolutional and pooling are generally assembled on the apex of each other to get out a greater number of representations of abstract features in mobility along the network. In CNNs, fully connected layers are essential for collecting global information, incorporating non-linearity, acquiring high-level inferences, providing final assumptions, and modifying the architecture for different applications.

### Training

An advanced, excellent model is not a better option to learn the parameters of models accurately and efficiently without an algorithm. However, for a task related to computer vision, a procedure of supervised training can be sufficient to understand an efficient model. ANNs and CNNs formally utilize learning algorithms to maintain their weights and biases which means their free parameters to achieve the desired output for the network. The most famous algorithm utilized for speeding up the evaluation of the gradient and for providing training to the neural networks is backpropagations^29^. Learning is traditionally estimated by certain loss function optimization. Generally, during the training of CNNs, some issues of overfitting occur. Overfitting is responsible for the poor performance of the networks in both cases of a small and large scale of datasets for training.

### Backpropagations

Backpropagation is a procedure that can be used for a neural network of feedforward, multilayer neural networks and is extensively used to train the system. This is the most famous and important technique utilized for the supervised training of CNNs. Backpropagation is performed by normalizing the value of weights internally by approximating the relationship of nonlinear types between the data of input and output. It works in two steps, which are training and testing. The sample inputs and the correction of classifications will be carried out in the training stage. The equation of backpropagation can be repeatedly implied to carry out gradients along every module, initiating from the output to the input.

## The architecture of the CNN model for image classification

**Figure 4:**
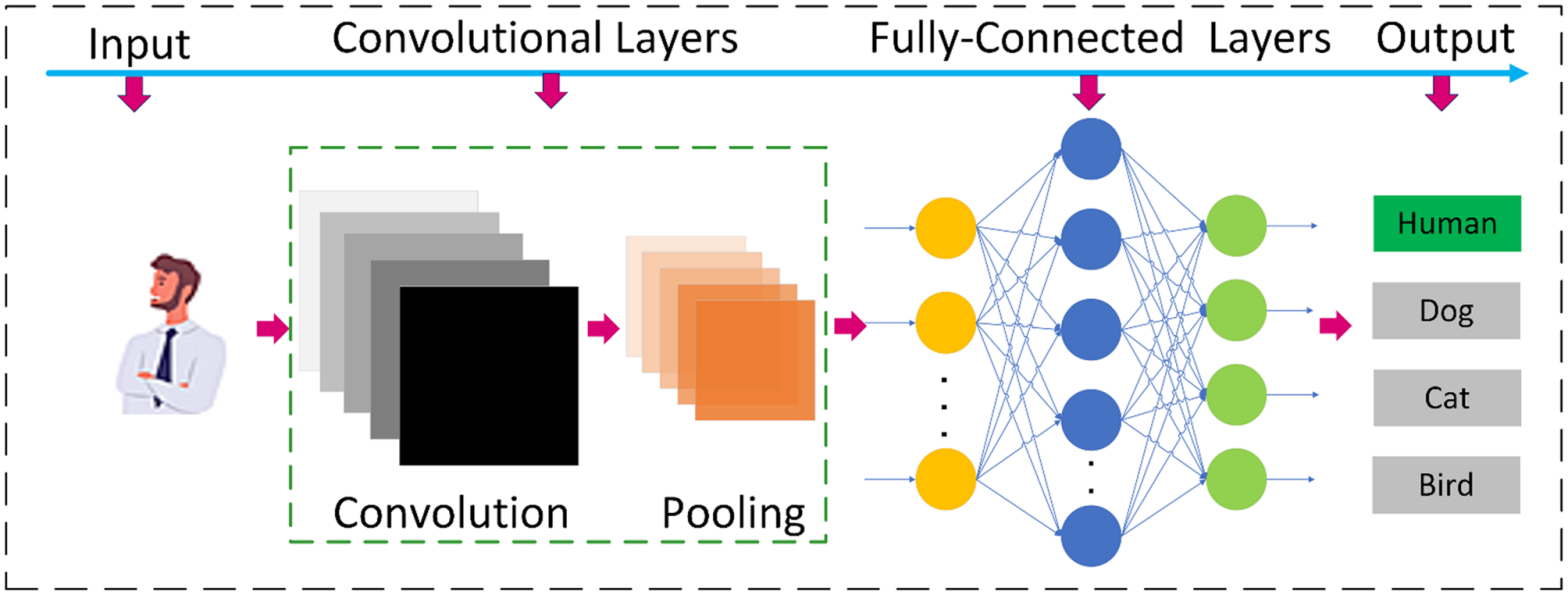
The complete internal structure of the CNN model for image classification.

CNNs are feedforward neural networks in which information only allows them to move towards output starting from their inputs stage. ANNs and CNNs both are biologically inspired. The human brain consists of a visual cortex, which has complex and simple cells of alternating layers, which inspires the architecture of CNN^30,31^. The architectures of CNN come in various patterns; although basically, they consist of pooling and convolutional layers, which are integrated into modules. As a typical feedforward neural network, similarly, either one or several fully connected layers emulate these modules. Finally, these modules are commonly assembled together to construct a deep model. Figure 4 demonstrates the standard structure of CNN for a task related to the classification of images or objects. An image of humans is input image data into the network, and later they are accompanied by various phases of convolutional and pooling layers. After that, descriptions from these performances feed one or several fully connected layers^32^. Lastly, the output comes from a fully connected layer in the form of a class label.

## Human activity recognition

**Figure 5:**
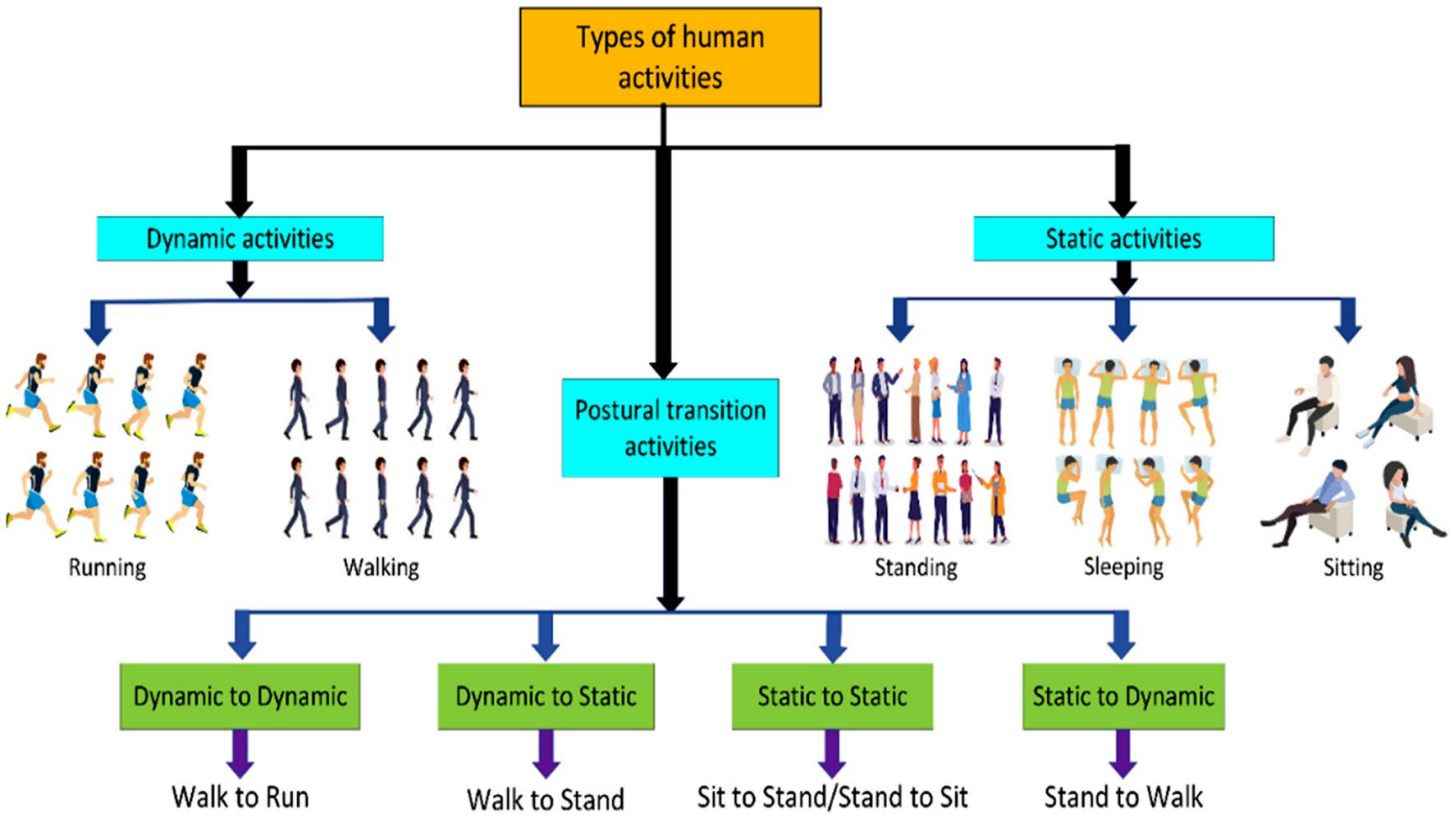
Types of human activities.

The automatic recognition and classification of actions taken by humans using information gathered from multiple sensors or other sources of input is known as HAR, and it represents a part of computer science and AI. While acquiring and analyzing confidential information on human behaviors, HAR has faced an array of constraints, including managing sensor noise, fluctuating activity trends, and maintaining privacy. HAR is a part of the pattern recognition problems that learn to recognize various human physical activities collected from different sources such as videos, images, and multiple sensor models. Recognition of different activities such as sleeping, running, walking, standing, sitting, etc. done by human beings in live videos is the most important type of computer vision task. HAR is widely utilized in various fields such as autonomous systems, context-aware computing, human-computer interactions, healthcare, automatic surveillance systems, human behavior analysis, ambient assistive living, robot learning, and many more^33^. Although various research and studies are still going on in this area, nevertheless it is an important and fascinating subject of study and research due to various complicated issues in the image collection from videos such as jumbled background, the viewpoint of cameras, size of humans, lighting variations, viewpoint variations, varied shapes, and occlusion. Human activities are mainly divided into three parts which are based on the position of the human body such as static, dynamic, and postural conditions. Where, standing, sleeping, and sitting are parts of static activities because human bodies remain steady during data collection, Running and walking are parts of dynamic activities because human bodies are not steady during data acquiring, and postural transition activities are in between static and dynamic. HAR is an essential component of pattern recognition, as it has a broad variety of real-world applications that influence our everyday lives, wellness, security, and performance. Various sensors, including gyroscopes, accelerometers, microphones, cameras, and many other data-gathering devices^34^, can be used to conduct HAR. The sensors can be integrated into wearable devices, smartphones, or permanent deployments. Figure 5 shows the types and list of human activities done by human beings on a daily basis in their lives.

## Research motivation and contribution of the research work

In recent decades, many researchers and technologists have been trying to develop an optimal HAR technique that will be applicable in various tasks such as robotics vision, medical assistance, surveillance, and many more. HAR plays an important role in interpersonal relations and human-to-human interactions. For a fluent and natural human-robot interaction, robots are required to have the potential of HAR techniques and can recognize the intentions of action and imitate the actions of people for a required response^35^. The ability of humans to identify another human activity is the most important part of study and research nowadays for scientific peoples of machine learning and computer vision. This research outcomes, many applications, including human-computer interactions, video surveillance, and human behavior recognition through robotics, demand a collective HAR system^36^. The proposed research contributes an appropriate addition to the state-of-the-art techniques by an enhanced rate of accuracy for the recognition system. When compared with conventional procedures, the proposed CNN-based HAR analysis provides an exclusive combination of automated learning of features, spatial-temporal modeling, resilience, and flexibility, resulting in an effective and advanced strategy for monitoring activities by humans. Thus, by exploiting CNN's automated extraction of features, spatial-temporal simulation, adaptability, and mobility, this proposed technique enhances the accuracy of the recognition system.

## Related work

In recent years, several researchers have been working in the field of computer vision, especially in human activity recognition to provide medical assistance to physically disabled people and hospital patients, surveillance systems, robot navigation, and many more. Poulose et al.^37^ proposed a technique for the HAR that utilizes the models of deep learning in the human image threshing machine. CNNs is a region-based model that is utilized for HAR, for the cropping and resizing of images, this work used a machine of facial image threshing, and for the classification of the image, a DL model is utilized. The rate of accuracy for HAR in this system is very high. Wu et al.^38^ describe a new technique for the recognition of multimodal gestures by using Deep Dynamic Neural Networks. A Hidden Markov Model-based framework of semi-supervised hierarchical dynamic is presented for contemporaneous segmentation and recognition of gesture where the observations of multimodal input are the skeleton joint data, depth, and RGB (red, green, and blue) images. This approach mostly utilized deep neural networks to learn representations of high-level spatiotemporal fitted to the modality of input data. Here, skeletal dynamics are handled by the Gaussian-Bernoulli Deep Belief Network, and the collections of depth and RGB images are handled and fused by using a three-dimensional CNN. Luwe et al.^39^ present a hybrid DL model for HAR-related tasks depending on the wearable sensor. This hybrid DL model consists of one-dimensional (1D) CNN integrated with bidirectional LSTM (long short-term memory). The rate of accuracy is 95.48% in this methodology. Zeng et al.^40^ present a technique for the automatic extraction of discriminative features for different HARs. This technique is based on CNN, which might gather scale invariance and local dependency of a signal, similarly as shown in the domain of speech and image recognition. Also, partial weight sharing is presented and implied to the signals coming from the accelerometer to achieve more enhancement. The rate of accuracy of this methodology is better than the state-of-the-art technique. Ha et al.^41^ presented a HAR technique through CNN by using multiple gyroscopes and accelerometer sensors. This study uses both partial and complete weight sharing to construct CNN-based models to enable them to learn from multi-sensor information. The multi-sensor data in this study are modality-specific properties and common features across techniques, which are subsequently gathered in the upper layers. As well as the multi-modal data is also responsible for training the common characteristics of the CNN-model through modalities. This proposed CNN model has higher and better performance with respect to the traditional approach of the HAR system. Ranjan et al.^42^ proposed an algorithm for simultaneous gender recognition, pose estimation, face detection, and landmark localization using deep CNN. The presented algorithm is called HyperFace. By using a separate CNN, the deep CNN intermediate layer gets fused and followed by an algorithm of multi-task learning that performs on the feature of the fused layer. This technique has significant impacts in the HAR field. Ding et al.^43^ proposed HAR based on a thermal infrared vision for providing individual surveillance at nighttime around parked aircraft. The proposed algorithm perfectly integrates the complete functionalities of this work as, a module of preprocessing in which a new structure of data is established to introduce information regarding human actions; a spatial feature extraction module based on CNN; a module for the extraction of temporal feature based on a triple layers of convolutional LSTM (Long Short-term Memory) network; and classification through two fully connected layers. The outcome of this research work gives a recognition rate of more than 96%. Gupta et al.^44^ present a HAR model based on the DL algorithm and utilizing wearable sensor data. This research work proposed a new hybrid model of deep neural networks. The hybrid model is combined with CNN and Gated Recurrent Unit (GRU) for HAR. The proposed model is successfully trained and validated, which gives higher accuracy than other deep neural network-based HAR models.

From the literature survey, it is clear that the CNN-based HAR technique has an important and remarkable effect on the recognition of activity. Thus, a greater number of CNN-based HAR approaches are required to enhance the performance, accuracy, and efficiency of the HAR. The future research trends in HAR could be the generation of synthetic data. In most methods and techniques utilized for the mining of data to learn efficiently the model of HAR, it is necessary to utilize a huge dataset for the model training. The data can have some issues such as class imbalance and intra-class variability because some activities occur over a longer time with respect to other activities. In a real-time case, a human might take a longer time for walking activity with respect to jogging activity. The duration of activities varies immensely for HAR such as bathing, sweeping, walking downstairs and upstairs, cleaning the house, walking, cooking, and so on. Thus, this time duration problem can be resolved by using synthetic data generation techniques^45^. Some researchers and technologists have already started to generate synthetic data for HAR tasks. The generation of synthetic data needs further research to estimate patterns of data that provide a better raw signal characterization for a different activity signal. Another interesting subject with the possibility of contributing to significant enhancements is the implementation of reinforcement learning (RL) methods for HAR. Advanced RL approaches for predicting prospective activity include proactive user support systems, assistive technology, healthcare, and many more. The real-time estimation of the dataset is gathered from sensors, sound, and images, among which algorithms can be highlighted and addressed soon based on Transfer Learning and Reinforcement Learning^46^.

## Proposed methodology

**Figure 6:**
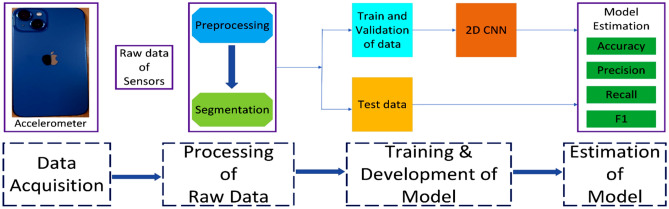
The framework of human activity recognition.

The classification challenge to recognize the daily activities of humans is known as human activity recognition (HAR). One of the main uses of wearable technology within medical surveillance is HAR, which requires constant tracking of everyday activities. The best methods for forecasting human actions using supervised machine learning (ML) are dependent on a constant supply of data from sensors. The research method taken in this study incorporates key techniques, such as collection or acquisition of data by using mobile sensors related to different activities of human, pre-processing of collected data, converting the collected raw data into their corresponding segments utilizing a sliding window of appropriate size, breaking the dataset towards training, validation and testing processes followed by the establishment of model utilizing several algorithms of DL, adapting the hyper-parameters and estimating the model's performance using several metrics of performance. This study used 2D CNN in the proposed model. These steps will ultimately assist in the supervised classification and recognition of activities done by a human from gathered datasets which can support medical practitioners in remote health monitoring of elderly or critically ill patients, surveillance at the border fencing for illegal migration or any terrorist activities, automatic navigation of mobile robots in the predefined environment, indoor surveillance for protecting our home from theft, and many more based on the movement of their body. Figure 6 represents the complete architecture of the proposed technique which shows the complete outline of this study through human activities classification using 2D CNN which means it is the complete framework of HAR.

In this study, we have taken WISDM datasets to analyze the videos that have various activities of humans such as sitting, running, walking, standing, and many more recorded from our phones. The WISDM dataset is taken in this study because it has the highest number of samples.

### Dataset description

There is a total of 1098207 samples that have been taken in the Wireless Sensor Data Mining (WISDM) dataset^47^. The WISDM dataset is unbalanced. This dataset has 36 subjects as experimental objects. Walking activity has the highest data of 38.6% while standing activity has only 4.4% data. This dataset is collected by keeping an android phone by the subjects in their pockets front leg. The sampling frequency of the accelerometer sensor utilized in this research work is 20 Hz. The smartphone is inbuilt with a motion sensor. There are six activities such as jogging/running, sitting, standing, walking, upstairs, and downstairs have been recorded. The collection of data was supervised by an expert to ensure the data quality.

**Figure 7:**
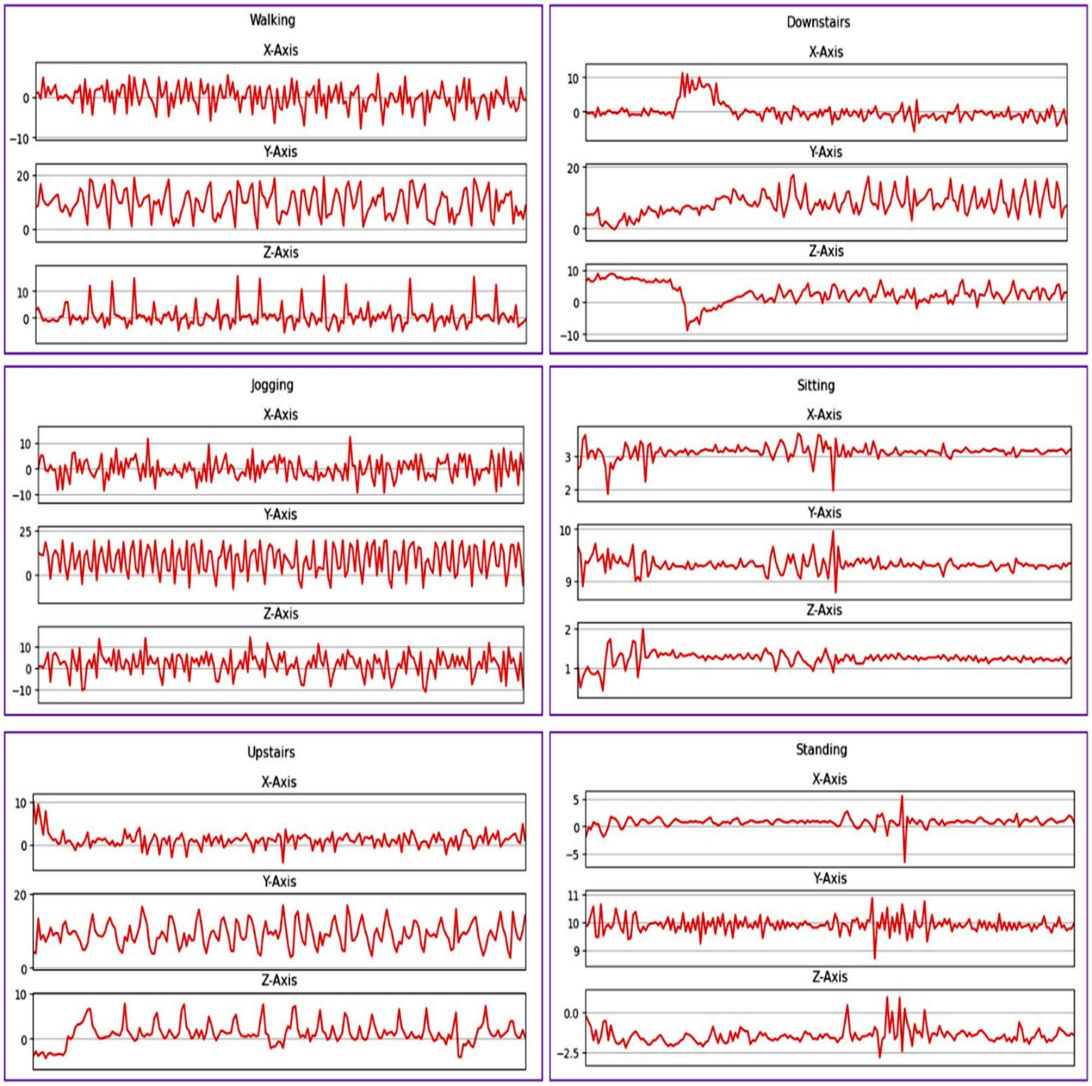
Acceleration waveform of each human activity.

Table 1 shows the complete details of the WISDM dataset utilized in this study. Table 2 provides the complete details of activities carried out during the collection of the WISDM dataset from 36 volunteers. Figure 7 illustrates the waveform of acceleration of each activity for 10 seconds with the ambition of showing the properties of the raw dataset on their corresponding X, Y, and Z axes.

**Table 1:** Information on the WISDM public dataset.

Dataset name	Sensor	Sampling rate	Activities	Volunteers	Samples
WISDM	Accelerometer	20 Hz	6	36	1,098,207

**Table 2:** Information on the WISDM public dataset.

Activities	Number of samples	Percentage
Walking	424,400	38.6
Jogging/Running	342,177	31.2
Upstairs	122,869	11.2
Downstairs	100,427	9.1
Sitting	59,939	5.5
Standing	48,395	4.4

### Preprocessing of data

To enhance the accuracy and feed the framework of the HAR model with an accurate dimension of data, the raw data gathered with accelerometer sensors are required to be pre-processed accordingly.

#### Linear interpolation

The above-given WISDM datasets are pragmatic, and the sensors used by the volunteers are wireless. Thus, some of the data might be missed during the procedure of data acquisition, and the missed data is generally designated with NaN/0. An algorithm of linear interpolation is used to overcome this problem.

#### Scaling and normalization

The direct utilization of large-scale data from the channels to train the models might lead to biases in the training. Therefore, input data is required to normalize in the range between 0 to 1 first before feeding them to the HAR model, accordingly, given below in Eq. (3)^48^:

3 $${Y}_{i}=\frac{{y}_{i}-{y}_{imin}}{{y}_{imax}-{y}_{imin}}(i =1, 2, 3, \dots , n)$$

where *n* is the number of channels, also, the minimum and maximum values of the *i*-th channels are *y*_*imin*_ and *y*_*imax*_ respectively.

#### Segmentation

This study utilized an end-to-end HAR model. The data sequence is considered as the input to the HAR model. This sequence of data is a type of short time series that is collected from the mobile sensor's raw data. It is the continuous recording process of data collection. A sliding window that has a 50% overlap rate is utilized to segment the extracted input data by the accelerometer sensors to ensure the temporal interconnection among the data points of each activity. The sliding window length is 128 readings per window, for the WISDM public dataset. It is best idea that our focus on the optimal size of the window was presented in an empirical and adaptive approach to creating better segments for every considered HAR^49^.

### Two-dimensional (2D) convolutional neural network

**Figure 8:**
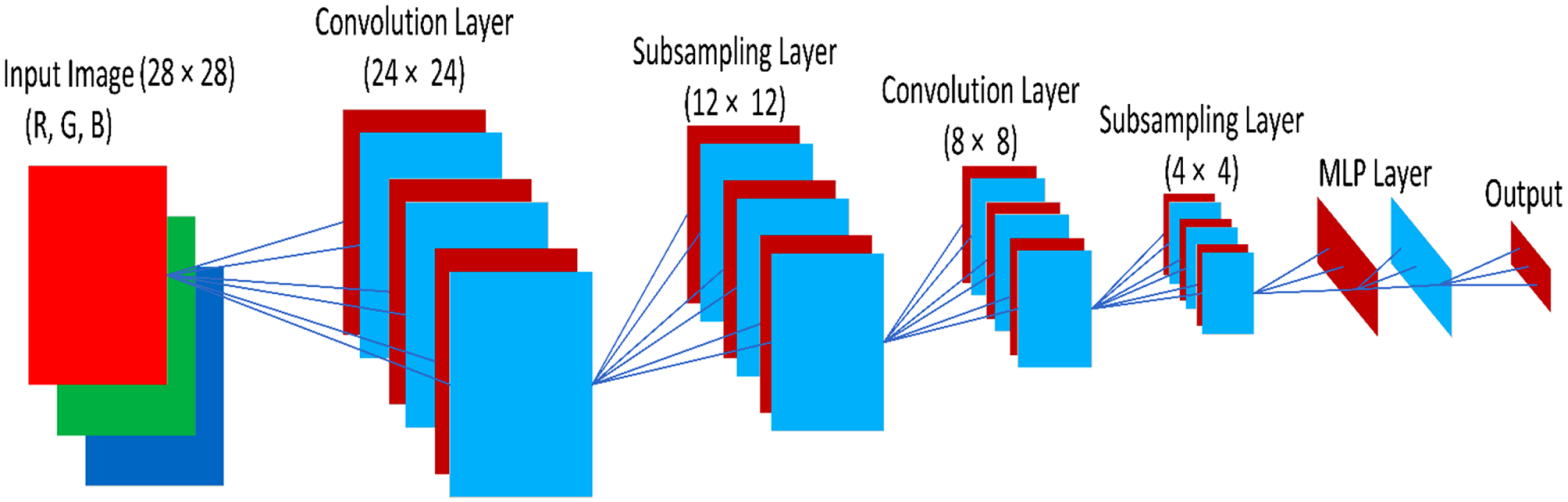
A basic illustration of a sample traditional CNN^50^.

2D CNN is an important type of CNN that has two types of tensors, where one tensor is considered as input and the second one is considered as output. The 2D convolutional layer is denoted by Conv2D, which makes a convolutional kernel that wraps with input layers that aid in generating an output tensor. The kernel is a mask or convolution matrix which might be utilized for edge detection, embossing, blurring, sharpening, etc. by making a convolution between an image and a kernel. During the experiment, we are using a total of 16 filters with each size (2, 2) in the first 2D CNN model, and for the second 2D CNN model, we are using a total of 32 filters with each size (2, 2). A common CNN architecture with (28 x 28)-pixel images is illustrated in Fig. 8. Within every convolutional layer, there are subsampling layers that eradicate the neurons' intended feature representations. After an adequate number of subsampling layers, the final subsampling layer approaches a scalar that represents a neuron. Following, CNNs have fully connected layers that retain an identical layout as MLPs.

**Figure 9:**
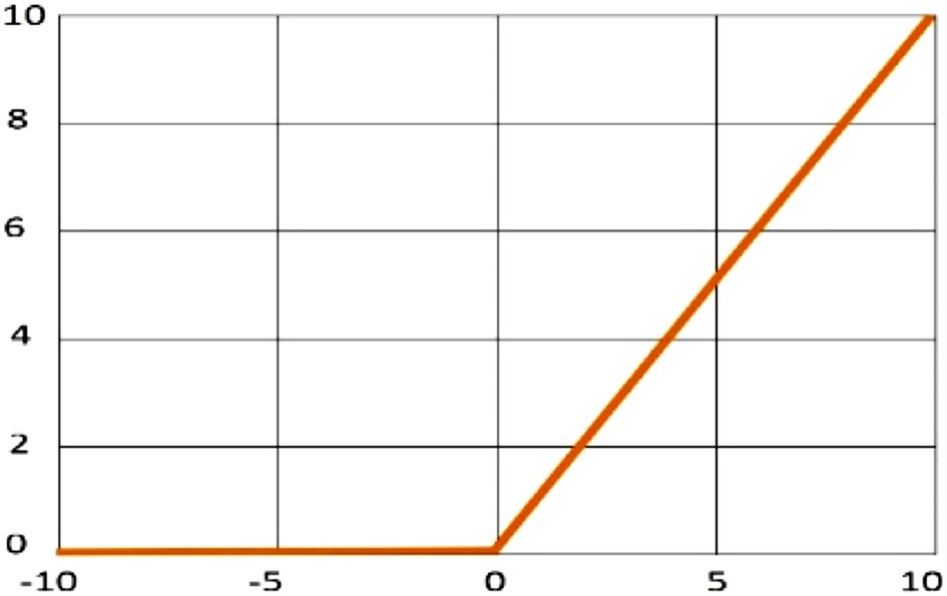
Graph of the ReLu activation function.

In this experiment Rectified Linear Unit (ReLu) activation function has been used. This activation function gives direct output if the value is positive, and the output will be zero if the value is negative. Equation (4) shows the ReLu activation function and is illustrated in Fig. 9^51^. A dropout layer is utilized to make a random set of hidden unit’s outward edges to zero at every training phase update. The value estimated by the dropout layer illustrates the probability of the dropped-out condition generated in the output layer. The dense layer is used in the model as the neural network of deeply connected 64 neurons. There is a total of 6 classes that have a total of 6 neurons in the output layer and it is dense. Also, the SoftMax activation function is used to change a real vector into a categorical probabilistic vector. The SoftMax activation function is generally utilized to activate the last layer in the classification step.

4 $$ R\left( z \right) \, = \, max \, \left( {0, \, z} \right), $$

where the ReLu activation function is *R(z)* and their corresponding pixel value is *z*.

### Training and validation

**Figure 10:**
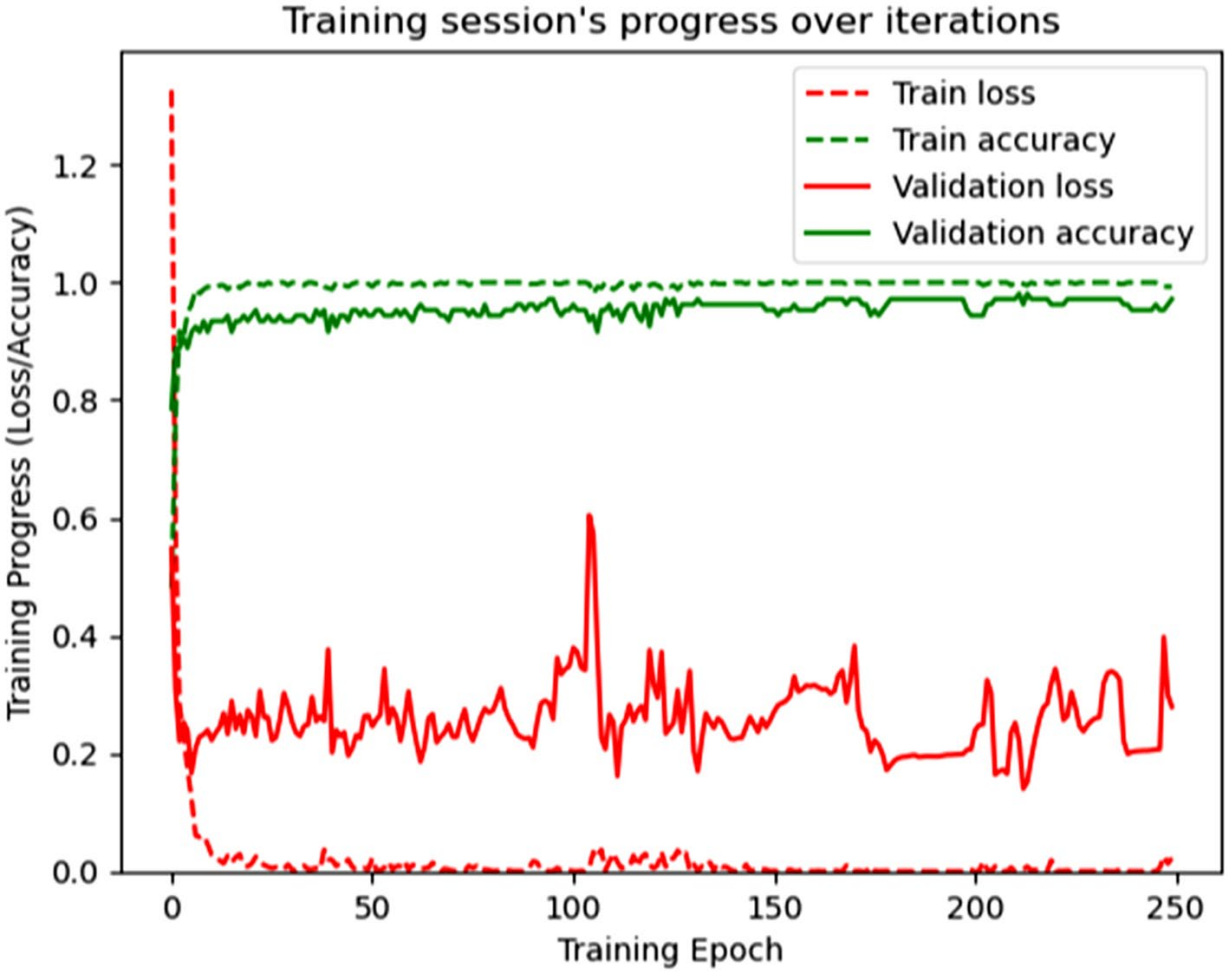
Progress of model training over the number of iterations.

In this study, a neural network of very high-level which is known as Keras was utilized to make the proposed network model. TensorFlow is a DL framework that can operate on Keras; as a result, TensorFlow executes on Keras. which is written in Python software. TensorFlow is also used in this experiment as a backend. The proposed model used a laptop for training and classification. The laptop has Windows 10 Enterprise version 22H2 with 1.70GHz, 8.00 GB RAM (Random Access Memory), and the operating system is equipped with 64-bit. A fully supervised technique is utilized to train the proposed HAR model. The weight and bias of every layer are initiated by the randomly collected values. Categorical cross-entropy is utilized to estimate the variance between the actual and probability distribution. Figure 10 demonstrates the progress of the proposed model training over the number of iterations.

### Output layers

In the proposed model, a SoftMax classifier and a fully connected layer are included in the output layer. Integration of fully connected layers in the last section of the proposed HAR model has a lot of benefits. Every node of the upper and fully connected layers is connected to provide integration of feature extraction from the upper layer in the system. SoftMax classifier transforms the outputs of the upper layer into a possible vector that belongs to the possible classes of samples.

**Figure 11:**
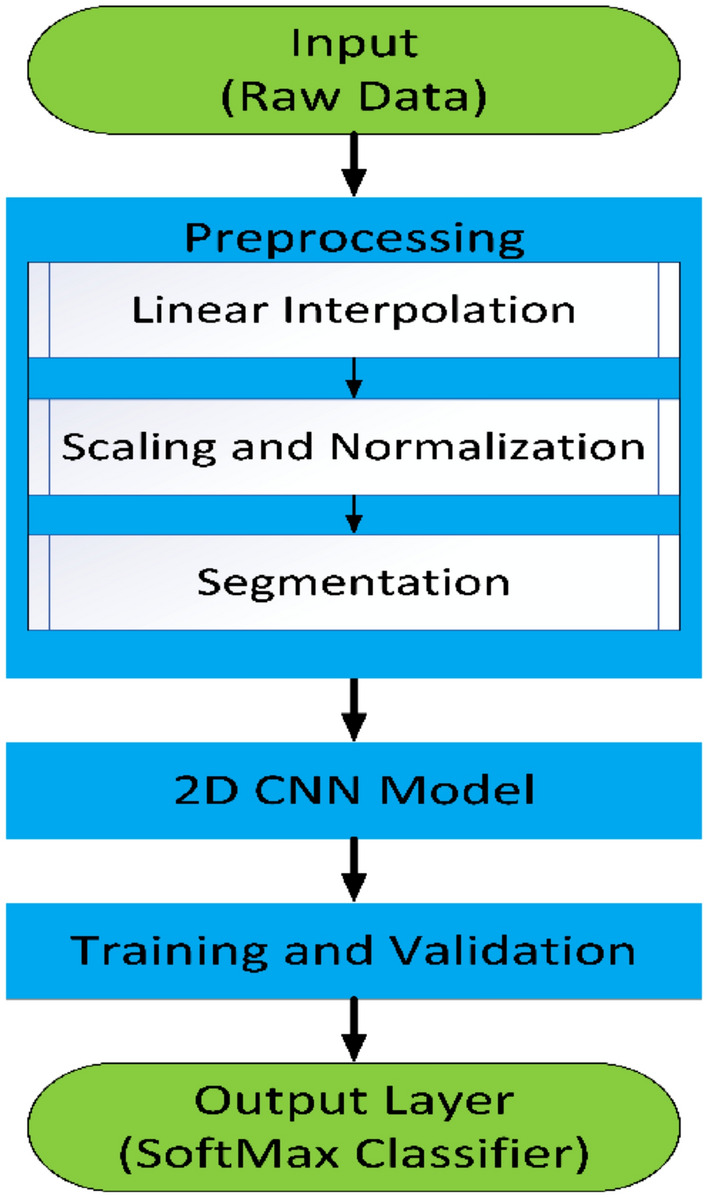
Flowchart of the proposed model.

The steps discussed above involving the proposed technique to recognize different human activities are illustrated in Fig. 11, which initially involves data collection from accelerometers, then goes through preprocessing procedures including linear interpolation, scaling and normalization, and segmentation. Next, the structure of CNN is shown in the flowchart. Subsequently, the training and validation phases are illustrated, where labeled data is used to teach the CNN how to recognize actions. Finally, in the output layer, the trained CNN model is used for the classification of activities.

## Experimental result and discussion

In this study, we used accelerometer data to train the proposed 2D CNN model, so that it can recognize the activities of humans. The WISDM public dataset is utilized to estimate the generalization efficiency and the accuracy of the proposed HAR model. This approach provides outstanding knowledge about the application of DL in the image classification process. We used a native Python file to read the WISDM public dataset and further, it is preprocessed to remove redundant information without losing necessary information. The input data is recorded regularly, and a standard technique is to utilize a predefined size of the sliding window for the segmentation of the output data coming from the sensors. As the WISDM dataset is an unbalanced distribution of data that means every activity has an independent number of examples. Standing has the lowest number of examples compared to Jogging and Walking. Therefore, if we use this dataset without balancing them then it will be overfitted and skewed towards Jogging and Walking. The hyperparameters and details of the system configuration which are utilized initially for the training and later for the testing of the proposed HAR model have been given in Table 3. A key stage in tuning the hyperparameters for the CNN-based HAR system is selecting the learning rates. To get optimal convergence and model accuracy while training a CNN to handle a HAR task, the learning rate needs to be carefully picked. Quickening training convergence and enhancing overall model efficiency are major advantages of the Adam optimizer's usage for CNN-based HAR systems. Adam's optimizer variable learning rates, which vary per parameter, are very helpful when employed with complicated and dynamic HAR datasets. The accuracy of the model can be improved by utilizing a greater number of epochs since this facilitates higher levels of convergence and can capture complex trends from the data, thus, we have selected a higher number of epochs.

**Table 3:** List of hyperparameters and details of system configuration.

System configuration	Description
Python version	2.9.1
TensorFlow version	2.9.1
Keras version	2.9.0
Pandas version	1.4.4
Numpy version	1.21.5
Optimizer	Adam
Learning rate	0.003
Loss	Categorical cross-entropy
Epochs	250
Processor	Intel(R) core (TM) i3-4005U

The proposed approach is tested by using the WISDM benchmark dataset within this section to determine the model's efficiency. The shape of the dataset contains a total of 343416 rows and 6 columns, where the walking activity contains 137375 rows, the jogging activity contains 129392 rows, the upstairs activity contains 35137 rows, the downstairs activity contains 33358 rows, the sitting activity contains 4599 rows, and the standing activity contains 3555 rows, respectively. The WISDM dataset skews for jogging and walking and can overfit when we use it straight. The standing activity has the lowest number of rows of all activities. Thus, to balance the dataset, we have taken 3555 rows from each activity. Thus, the total number of rows during the experiment is 21330 (3555x6) and 4 columns. Now, we will divide this aggregate number of samples for total activities with the quantity of samples within each frame in a sequence which is 40 to get the number of frames. So, the number of frames will be approximately 532. In this experiment, test data is 20% of the total data and the remaining 80% of the data is considered as the training set. Table 4 illustrates the number of cases of the training set of data and the test set of data considered in this experiment from the input dataset after segmentation.

**Table 4:** Instances of the WISDM public dataset for the study.

Set of data	Number of data
Training sample	425
Test sample	107

The model is compiled and trained with the above-estimated training data over 250 epochs or iterations. We utilized parameters such as accuracy score, precision score, recall score, and F1 score for the estimation of model efficiency and performance. The following given equations (5), (6), (7) and (8) define these parameters consecutively^52^.

5 $$Accuracy=\frac{TP + TN}{TP + TN + FP + FN},$$

6 $$Precision=\frac{TP}{TP+ FP},$$

7 $$Recall=\frac{TP}{TP + FN},$$

8 $$F1=\frac{2 \times Recall \times Precision}{Recall + Precision} ,$$

where true positive, true negative, false positive, and false negative are denoted by *TP, TN, FP*, and *FN* consecutively.

**Figure 12:**
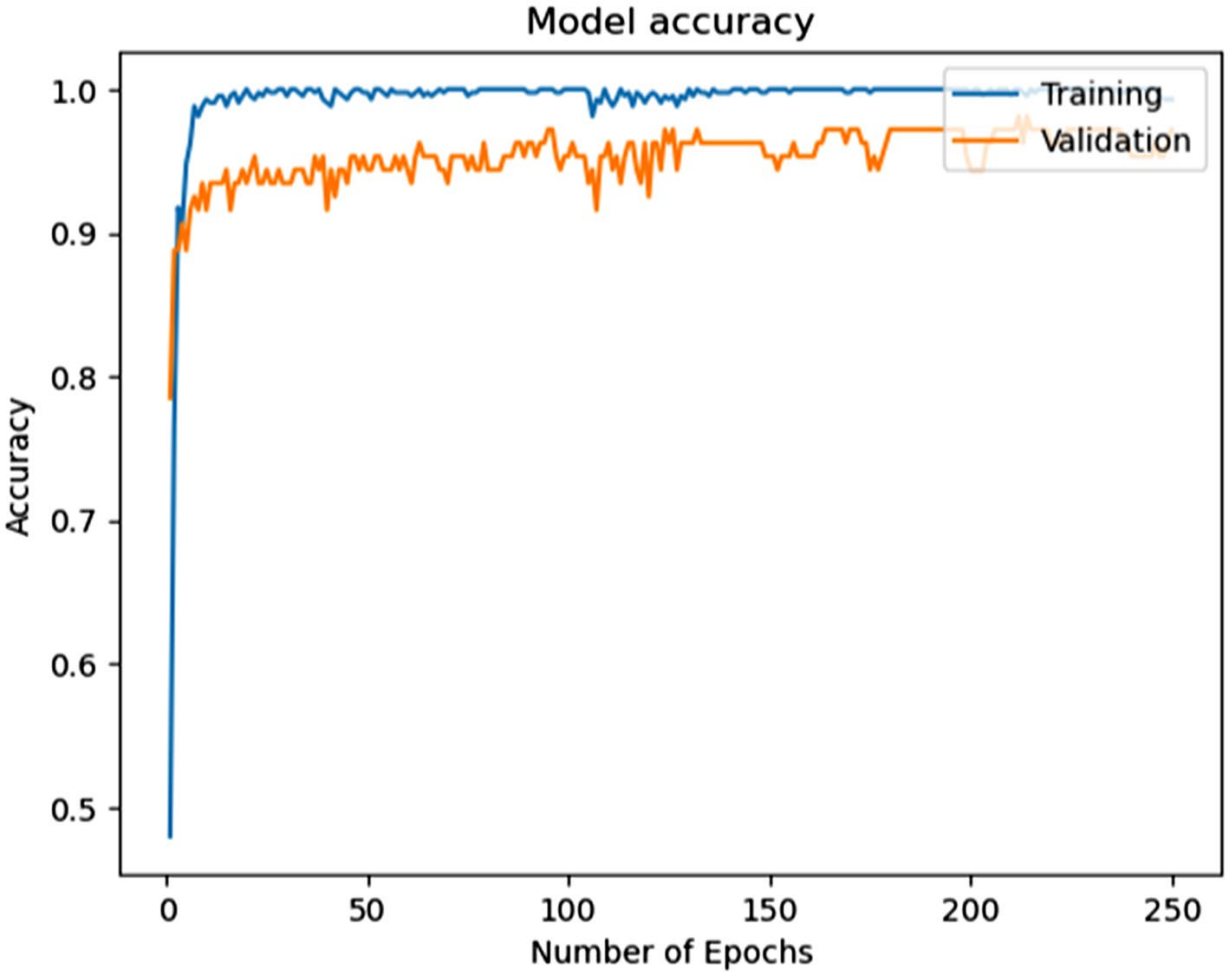
Accuracy in the model training and validation over the number of iterations.

**Figure 13:**
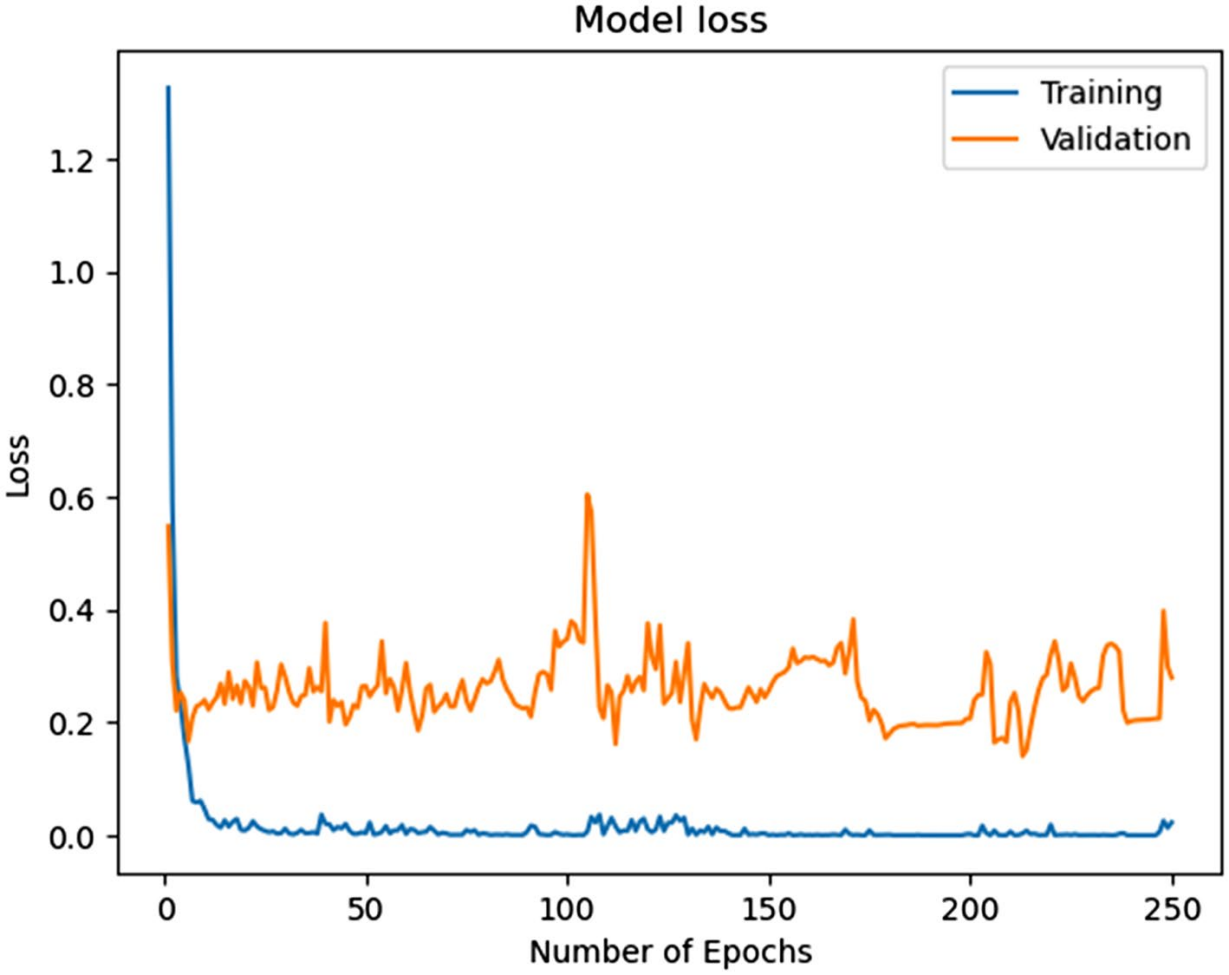
Losses in the model training and validation over the number of iterations.

This model is capable of automatically obtaining hierarchical characteristics using unprocessed data from sensors to enable precise activity classification. After the successful modeling of the proposed HAR model to recognize activities with better accuracy and validation, we have plotted the model accuracy graph containing training accuracy and validation accuracy, and the model loss graph containing training loss and validation loss over the number of iterations, which are illustrated with these details in the Figs. 12 and 13.

**Figure 14:**
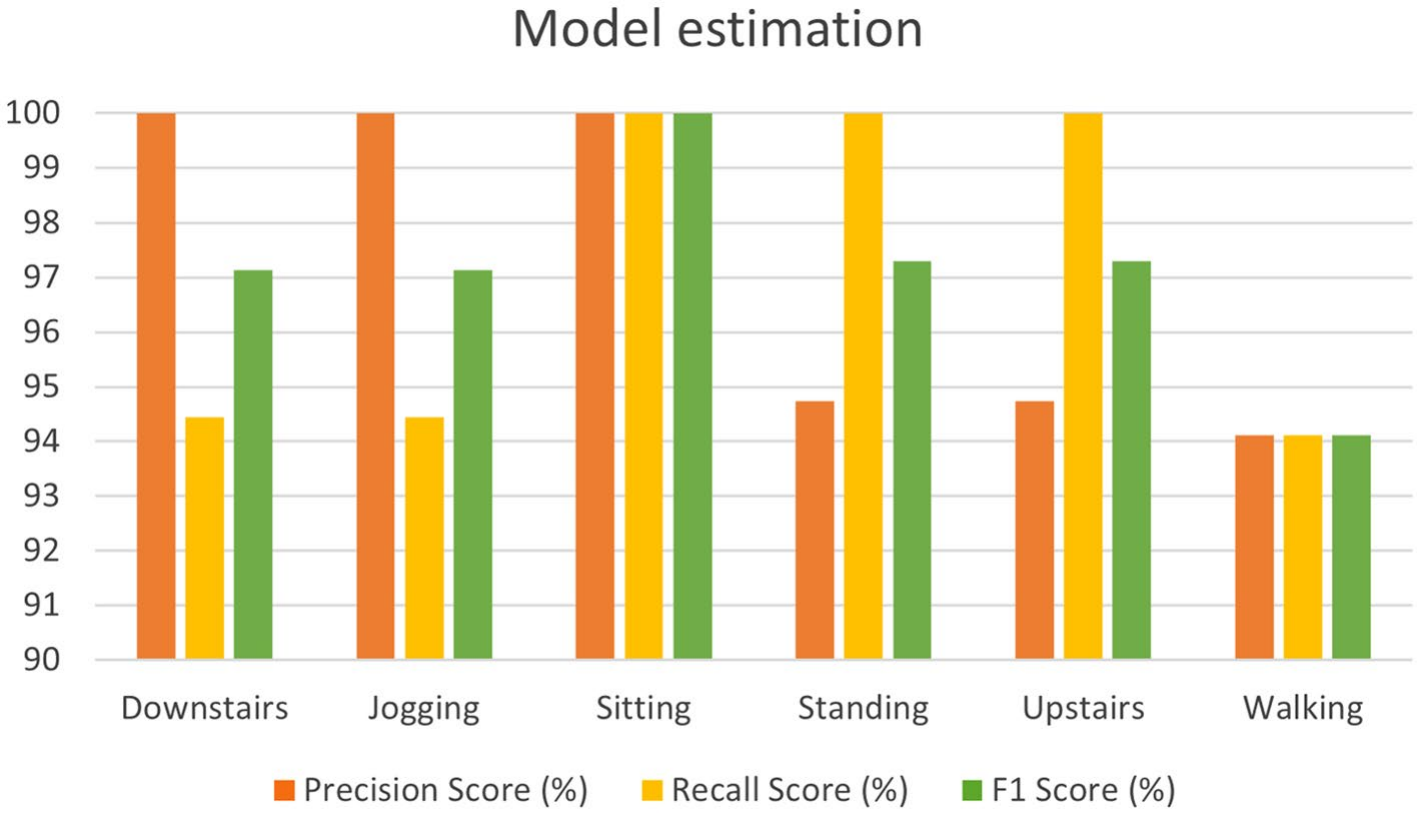
Illustration of different parameters for the model estimation.

The training and validation accuracy scores are very high during the performance of the model, which can be seen in Fig. 12. The training and validation loss scores are very low during the performance of the model, which can be seen in Fig. 13. After the training and validation of the proposed model, we evaluated different parameters for the model estimation. Figure 14 shows the evaluated value of the precision score, recall score, and F1 score for each activity.

**Figure 15:**
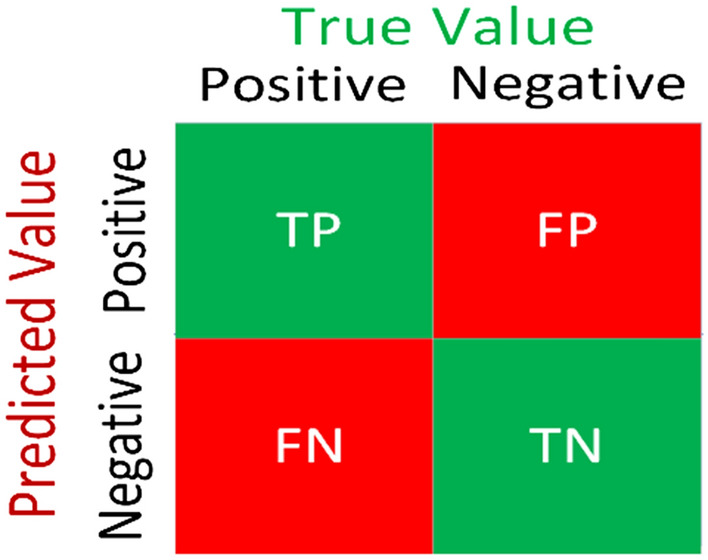
Confusion matrix of two classes.

Confusion matrices are a special kind of table that is generally used in the estimation of the performance index for the model through a collection of test data that has known true values. Rows of the confusion matrix present the prediction values of in-stances while columns of the confusion matrix present the true value of the instances or vice versa. The confusion matrix diagonal represents the true predictions while others show the errors. Thus, it is quite easy to check errors in the model by checking the table visually. Figure 15 represents a typical confusion matrix for two classes.

**Figure 16:**
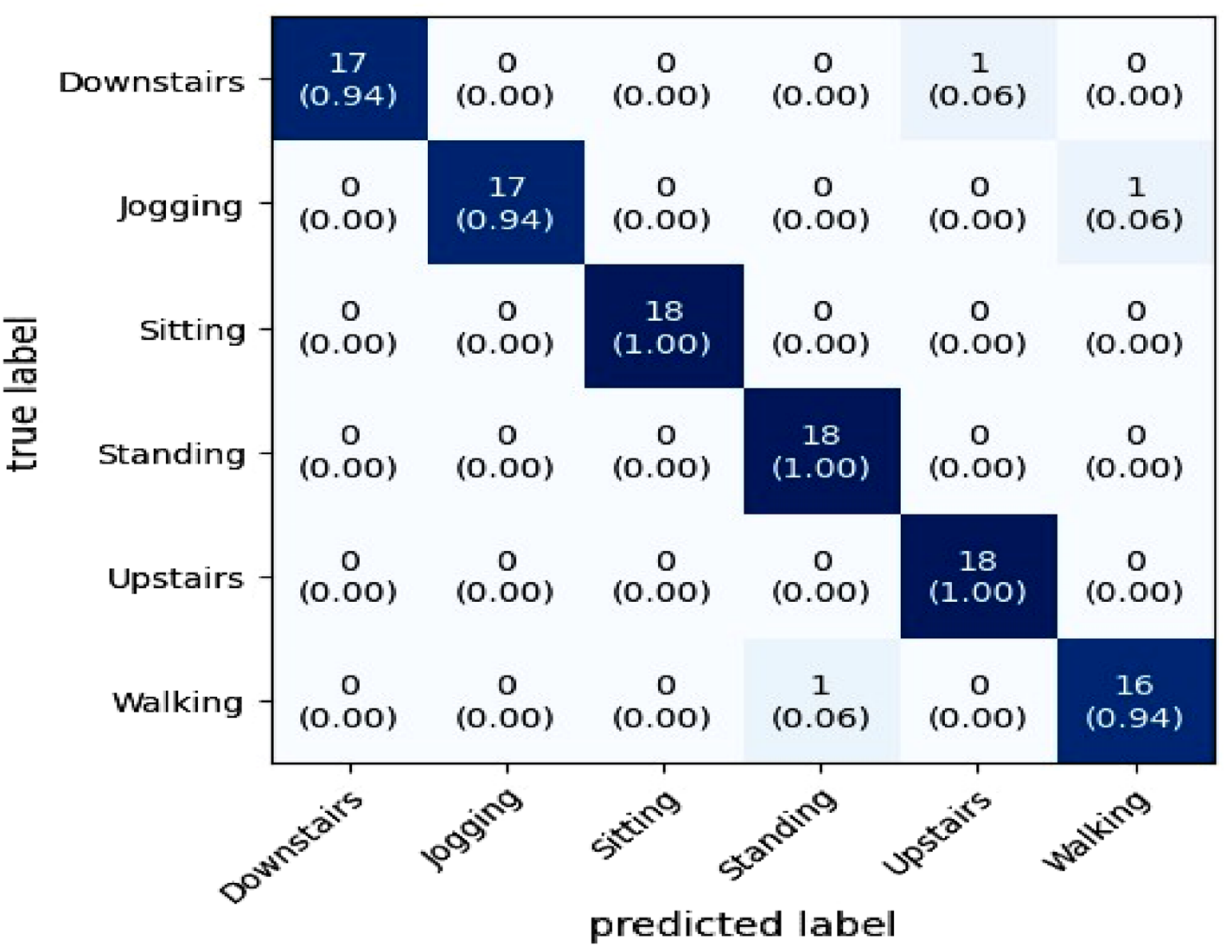
Confusion matrix for the performance estimation of the proposed study model.

Employing the optimal CNN features, the accuracy of this CNN-based HAR technique is investigated. Finally, we have plotted the normalized confusion matrix of the proposed study for the HAR model, which is provided in Fig. 16. The diagonal of the plotted confusion matrix represents the proposed model performance accuracy for different human activities. It is observed through the confusion matrix that when recognizing human activities, walking, jogging, and downstairs activities are seen to have very little confusion; however, sitting, standing, and upstairs activities have no confusion. Here, the rate of accuracy is 100% for the sitting, upstairs, and standing activities while it is 94% for the case of downstairs, walking, and Jogging.

This CNN-based HAR technique is superior to other contemporary methods mainly because it can automatically learn hierarchy attributes, appropriately structure spatial-temporal details, adjust for sensor variation, employ transfer learning, connect hybrid information, and acquire information from collaborative methods. Thus, collectively these attributes work together to deliver the greater recognition accuracy achieved by the proposed CNN-based HAR systems. The overall accuracy and losses of the proposed study is provided in Table 5.

**Table 5:** Overall performance indexes for the proposed study.

Model name	Accuracy (%)	Loss (%)
2D CNN	97.20	2.80

**Figure 17:**
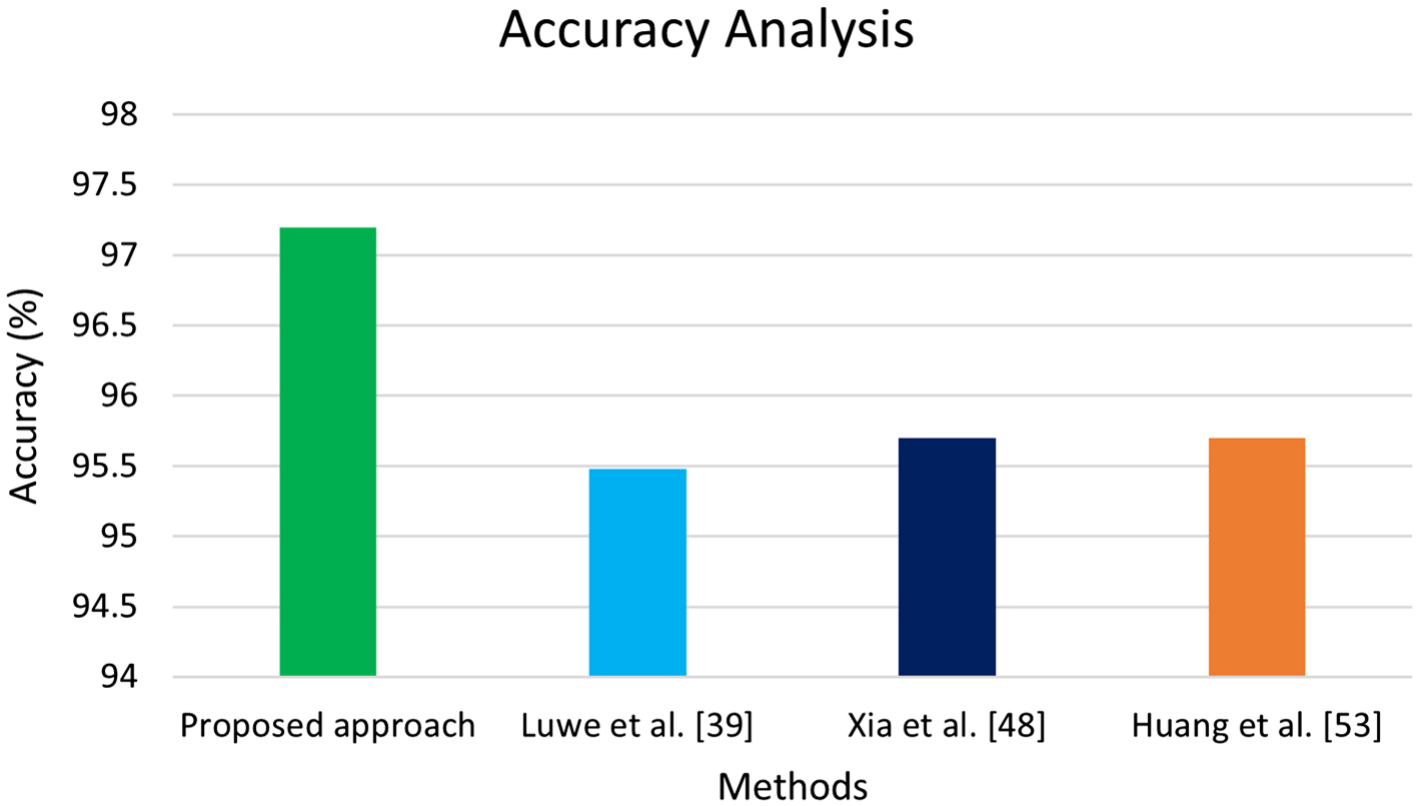
Comparative analysis between the proposed approach and state-of-the-art approaches.

The comparative analysis among different state-of-the-art techniques, including Luwe et al.^39^, Xia et al.^48^, and Huang et al.^53^, based on the CNN model for HAR using the WISDM dataset and the proposed approach, has been discussed in detail in Fig. 17. The rate of accuracy for the proposed methodology is higher than other methods illustrated in Fig. 17.

## Conclusion and future perspectives

The proposed 2D CNN-based HAR model can more precisely recognize human activity from raw input data collected by sensors. The CNN-based image classification model plays a vital role in the domain of pattern recognition or computer vision. In recent days, applications of HAR in numerous domains such as health monitoring systems, surveillance in industries, traffic control systems, military surveillance, robotics navigation systems, etc. are growing rapidly due to technological advancement in CNN and mobile sensor technology. Also, nowadays it is easier and more reliable to collect data due to availability and advances in the sources of data and process of data acquisition advancement, but it is still a challenging and tough task to make an efficient and reliable model for HAR. This article provides a comprehensive overview of CNN's principle of working toward image classification. We analyzed the role of a 2D CNN-based model for HAR through an experimental study by using the WISDM public dataset. The rate of accuracy, in this case, the study is more than the techniques utilized in the state-of-the-art models of the activity recognition system. This study is more concise and better than previous studies depending on the state-of-the-art, which will provide the fundamental ideas about CNN, HAR, and their relation for image classification tasks to the early-stage researchers. This CNN-based HAR strategy has a chance to significantly improve a variety of everyday uses, including reliable detection of falls that might secure lives, sports, and fitness via specific exercise guidance and efficiency assessment, improving performance during training, and facilitating immediate medical care for senior citizens.

The acquisition of data, preprocessing, handling of complicated tasks, and hardware constraints all provide difficulties for HAR. The feasible field for research in HAR will be fusing and processing data from miscellaneous devices. Although it is the situation that originated using the collected data through a single device does not execute excellently with the other devices. It takes place because of the heterogeneous issues among the devices. Heterogeneous properties are available among several smartwatches and mobile devices. Estimating deep learning and cross-domain transfer to shelter heterogeneity in the trained models are efficient further directions in HAR. The application of HAR in a variety of tasks provides exceptional advantages, but existing HAR models have various open challenges and limitations that should be addressed. Thus, there are several issues related to the collection of data, preprocessing of data, misalignment of activities, complex activities, and hardware that must be addressed carefully to enhance the model performance, model efficiency, and model accuracy. Furthermore, we will consider analyzing and comparing the HAR through the YOLO algorithm and RL by utilizing this study. This research will provide better insight to the early-stage researcher and experienced researcher about the CNN-based HAR technique.

## Data Availability

Correspondence and requests for materials should be addressed to the corresponding author on reasonable request. The datasets analyzed during the current study are publicly available in the WISDM Lab repository (https://www.cis.fordham.edu/wisdm/dataset.php).

## Code availability

The computer code used to support the findings of this study are made publicly available at; https://github.com/raj91ravi/HAR-using-CNN.
